# Controllable Construction of Aptamer-Modified Fe_3_O_4_@SiO_2_-Au Core-Shell-Satellite Nanocomposites with Surface-Enhanced Raman Scattering and Photothermal Properties and Their Effective Capture, Detection, and Elimination of *Staphylococcus aureus*

**DOI:** 10.3390/molecules29153593

**Published:** 2024-07-30

**Authors:** Yongdan Wang, Shengyi Wang, Yuhui Zou, Yuze Gao, Boya Ma, Yuhan Zhang, Huasong Dai, Jingmei Ma, Wenshi Zhao

**Affiliations:** 1 School of Foreign Languages, Jilin Normal University, Siping 136000, China15898157806@163.com (Y.Z.); 2 Key Laboratory of Functional Materials Physics and Chemistry of the Ministry of Education, Jilin Normal University, Changchun 130103, China; 15943440659@163.com (S.W.); 19990599393@163.com (Y.G.); 13664198753@163.com (B.M.); zhangyuhan193@163.com (Y.Z.); 15948835770@163.com (H.D.); jlsfdxmjm@163.com (J.M.)

**Keywords:** Fe_3_O_4_@SiO_2_-Au core–shell–satellite nanocomposites, aptamer, SERS, photothermal therapy, *Staphylococcus aureus*

## Abstract

The early monitoring and inactivation of bacteria are of crucial importance in preventing the further spread of foodborne pathogens. *Staphylococcus aureus* (*S. aureus*), a prototypical foodborne pathogen, is widely present in the natural environment and has the capability to trigger a range of diseases at low concentrations. In this work, we designed Fe_3_O_4_@SiO_2_-Au core–shell–satellite nanocomposites (NCs) modified with aptamer for efficient capture, high-sensitivity surface-enhanced Raman scattering (SERS) detection, and photothermal therapy (PTT) against *S. aureus*. Fe_3_O_4_@SiO_2_-Au NCs with tunable Au nanocrystal nanogaps were prepared. By combining the finite-difference time-domain (FDTD) method and experimental results, we studied the electric field distribution of Fe_3_O_4_@SiO_2_-Au under different Au nanogaps and ultimately obtained the optimal SERS substrate FSA-60. The modification of aptamer on the surfaces of FSA-60 could be used for the specific capture and selective detection of *S. aureus*, achieving a detection limit of as low as 50 cfu/mL. Furthermore, Apt-FSA-60 possessed excellent photothermal properties, demonstrating the strong photothermal killing ability against *S. aureus*. Therefore, Apt-FSA-60 is a promising high-sensitivity SERS substrate and efficient photothermal agent and is expected to be widely applied and promoted in future disease prevention and treatment.

## 1. Introduction

Diseases caused by foodborne pathogens pose a serious threat to the health and safety of the population [1]. As a representative of Gram-positive bacteria, *Staphylococcus aureus* (*S. aureus*) is a common foodborne pathogen that causes various serious diseases such as folliculitis, pneumonia, endocarditis, and gastritis [2,3]. Given the widespread presence of *S. aureus* in the natural environment, especially in wastewater, it easily causes food contamination [4]. Therefore, pollution should be fundamentally prevented. There is an urgent need to develop a new sensitive detection strategy to achieve the early detection of bacteria in wastewater. At present, the traditional methods for detecting *S. aureus* mainly include the microbial culture method, the fluorescence polymerase chain reaction (PCR) method, and the isothermal amplification method [5,6]. However, these methods require expensive equipment and complicated operations, which wastes time and requires professional operators [7,8]. In addition to effective detection and identification, the efficient killing of *S. aureus* is equally important. However, most current research has only concentrated on single functional bacterial detection or killing platforms. Therefore, developing a multifunctional platform that integrates bacterial recognition, detection, and killing functions is an important means to achieve the prevention and treatment of *S. aureus*.

Surface-enhanced Raman scattering (SERS) has become a promising and important technology in food analysis and bioassay because of its high sensitivity and selectivity, unique spectral fingerprinting, photobleaching, and non-invasive data acquisition [9,10,11,12]. Recently, researchers have displayed a keen interest in the utilization of SERS detection for the identification of pathogenic microorganisms [13]. The SERS method is capable of amplifying the Raman signal of molecules adsorbed on nanoscale metal substrates by multiple orders of magnitude, thereby obtaining the fingerprint spectrum of the analyte [14,15,16]. Especially in some closely spaced nanoparticle (NP) aggregation systems, the surface plasmon coupling between NPs forms a significant electromagnetic field enhancement, called a SERS hotspot [17]. Research shows that Au nanocrystals with good biocompatibility and strong localized surface plasmon resonance (LSPR) properties can generate sensitive SERS signals, making them suitable as SERS matrix materials for bacterial detection [18]. In addition, due to the relatively low concentration of bacteria in actual food, it is necessary to preconcentrate the target bacteria in such scenarios to obtain high-intensity SERS spectra of target molecules.

Fe_3_O_4_ magnetic nanocrystals have garnered extensive application in biomedical fields owing to their advantages, including facile synthesis, effortless surface modification, exceptional stability, and unique superparamagnetic properties [19,20,21]. These characteristics render them a prime candidate for isolating target bacteria. Unfortunately, naked Fe_3_O_4_ nanocrystals are prone to oxidation and aggregation in the air, and the introduction of a non-magnetic layer (such as SiO_2_) can inhibit this phenomenon [22]. Meanwhile, in order to accurately identify *S. aureus* in complex solutions, it is necessary to introduce recognition molecules with significant binding affinity and specificity [23]. Compared with other recognition elements, aptamer (Apt) possesses advantages like faster synthesis, lower production costs, high chemical stability, and a strong specific recognition ability [24,25]. Therefore, aptamer-modified Fe_3_O_4_@SiO_2_-Au core–shell–satellite NCs as the SERS substrate can be used for capturing and separating target bacteria in complex solutions under the action of magnetic fields, promising a highly specific and sensitive detection method.

Moreover, the efficient killing of pathogens is also urgently needed [26]. In current antibacterial strategies, photothermal therapy (PTT) has gained widespread recognition among researchers as a sterilization method with a short treatment time, minimal damage to normal tissues, and no drug resistance [27,28,29]. Under near-infrared (NIR) light irradiation, PTT converts the light energy absorbed by photothermal agents (PTAs) into heat and causes the thermal denaturation of proteins and DNA through rapid heating, leading to irreversible bacterial damage without producing toxic byproducts [30,31]. Fortunately, Fe_3_O_4_@SiO_2_-Au SERS substrates not only enable the highly sensitive SERS detection of bacteria but are also considered an ideal PTA, wherein Au nanocrystals demonstrate high photothermal conversion efficiency [32]. Moreover, introducing aptamer into Fe_3_O_4_@SiO_2_-Au NCs endows them with extraordinary selective recognition and enrichment abilities, thereby more effectively inactivating bacteria.

**Scheme 1:**
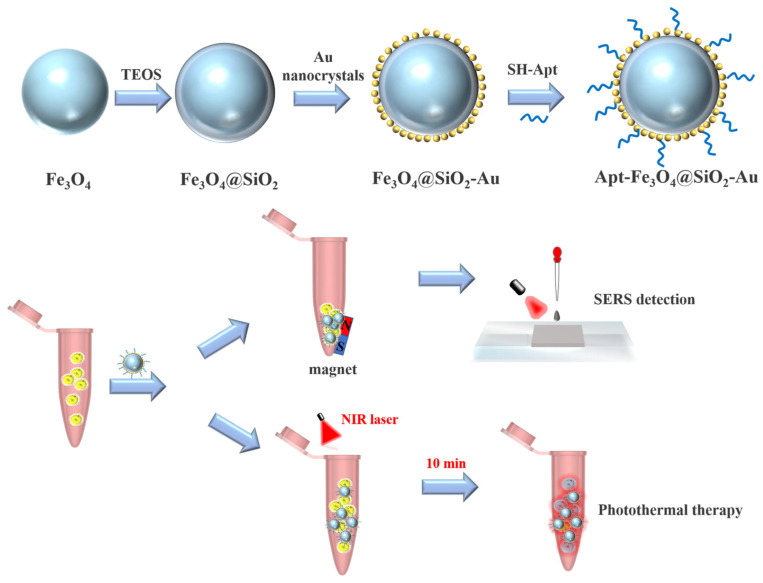
Synthetic route of Apt-Fe_3_O_4_@SiO_2_-Au and their SERS detection and PTT treatment for *S. aureus*.

Based on the above-mentioned inspirations, a novel multifunctional Apt-Fe_3_O_4_@SiO_2_-Au system that combines magnetic separation, specific capture, ultrasensitive SERS detection, and humorous photothermal inactivation was proposed. Fe_3_O_4_@SiO_2_ nanocrystals with magnetic-assisted separation capability were chosen for magnetic support materials, and Au nanocrystals with high SERS and excellent photothermal properties were loaded on their surface to serve as the SERS substrate and photothermal agent. By analyzing the correlation between the nanogap of adjacent Au nanocrystals and SERS performance through the finite-difference time-domain (FDTD) method and the use of 4-MBA molecules in experiments, a potential SERS enhancement mechanism was discussed. After combining the optimized FSA-60 with aptamer, it was used to detect *S. aureus*. Furthermore, the photothermal performance of Apt-FSA-60 was tested for the PTT of *S. aureus* and the photothermal inactivation mechanism was revealed. The synthesis route of Apt-Fe_3_O_4_@SiO_2_-Au and the schematic diagram of *S. aureus* SERS detection and photothermal inactivation experiments are shown in Scheme 1. This magnetic core–shell–satellite Apt-FSA-60 provides inspiration for the customized design of bacterial sensitive detection and efficient inactivation platforms.

## 2. Results and Discussion

### 2.1. Characterization and Optimization of Fe_3_O_4_@SiO_2_-Au NCs

**Figure 1:**
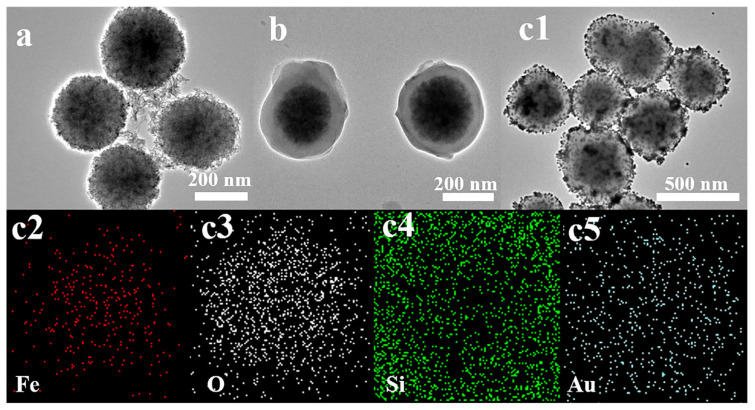
TEM image of (**a**) Fe_3_O_4_ nanocrystals, (**b**) Fe_3_O_4_@SiO_2_ nanocrystals, (**c1**) TEM image of Fe_3_O_4_@SiO_2_-Au NCs, and (**c2**–**c5**) EDS elemental mapping images (Fe, O, Si, and Au).

Figure 1 shows the TEM images of the synthesized Fe_3_O_4_ nanocrystals, Fe_3_O_4_@SiO_2_ nanocrystals, and Fe_3_O_4_@SiO_2_-Au NCs. From Figure 1a, the average size of Fe_3_O_4_ nanocrystals is uniform, with a size of approximately 205 nm. Figure 1b shows that Fe_3_O_4_ nanocrystals are encapsulated by an amorphous SiO_2_ layer with a thickness of 20 nm, ultimately forming an Fe_3_O_4_@SiO_2_ core–shell structure. After loading Au seeds onto Fe_3_O_4_@SiO_2_ nanocrystals through electrostatic attraction, numerous Au nanocrystals with a diameter of 14 nm randomly and uniformly adhere to Fe_3_O_4_@SiO_2_, which can be confirmed from TEM and EDS element mapping images in Figure 1c1–c5.

**Figure 2:**
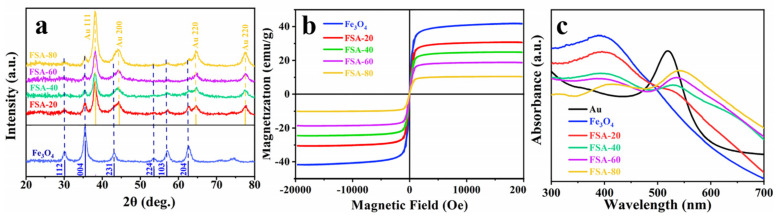
(**a**) XRD patterns of Fe_3_O_4_ nanocrystals and Fe_3_O_4_@SiO_2_-Au NCs (FSA-20, FSA-40, FSA-60, and FSA-80); (**b**) magnetic hysteresis loops of Fe_3_O_4_ nanocrystals, FSA-20, FSA-40, FSA-60, and FSA-80; (**c**) UV-Vis spectra of Fe_3_O_4_ nanocrystals, Au nanocrystals, FSA-20, FSA-40, FSA-60, and FSA-80.

During the SERS detection process, factors such as the content and distribution of noble-metal nanocrystals in the SERS-active substrate significantly influence the overall SERS performance. To obtain the highly sensitive SERS substrate, we optimized the loading amount of Au nanocrystals on Fe_3_O_4_@SiO_2_ nanocrystals. We characterized the structure of Fe_3_O_4_ and Fe_3_O_4_@SiO_2_-Au NCs with different Au loadings using the XRD method in Figure 2a. The diffraction peaks can be observed at 30.3, 35.6, 43.4, 53.4, 57.3, and 62.8°, corresponding to the reflections of (220), (311), (400), (422), (511), and (440) crystalline planes of Fe_3_O_4_, respectively (JCPDS card NO. 19-0629) [33]. After successfully loading Au nanocrystals onto the surface of Fe_3_O_4_@SiO_2_ nanocrystals, the four new peaks appearing at 38.2°, 44.3°, 64.5°, and 77.5° can be indexed to the (111), (200), (220), and (311) crystalline plane of Au (JCPDS card NO. 04-0784) [34]. With the addition of Au, the diffraction peak intensity of Fe_3_O_4_ nanocrystals decreases, while the Au diffraction peak intensity increases, indicating a gradual increase in the adsorption amount of Au on Fe_3_O_4_@SiO_2_ surfaces. Magnetic properties were investigated in Figure 2b. All of the NPs exhibit superparamagnetic properties, which not only prevent aggregation but also enable magnetic NPs to rapidly redisperse without a magnetic field. Magnetic saturation (Ms) values are 41.6, 30.7, 24.8, 18.7, and 10.4 emu/g for Fe_3_O_4_ nanocrystals FSA-20, FSA-40, FSA-60, and FSA-80, respectively. Compared to Fe_3_O_4_ nanocrystals, the decrease in the Ms value of Fe_3_O_4_@SiO_2_-Au NCs is mainly attributed to the coating of non-magnetic materials, including SiO_2_ and Au. Even though the Ms value of FSA-80 is low, it can be separated from the solution within about 20 s with the help of an external magnetic field. Figure 2c displays the UV-Vis spectra of Fe_3_O_4_ nanocrystals, Au nanocrystals, FSA-20, FSA-40, FSA-60, and FSA-80. For Fe_3_O_4_ nanocrystals, a broad absorption spectrum is noticeable, which is because Fe_3_O_4_ nanocrystals are black and will absorb at all wavelengths [35]. Au nanocrystals show a characteristic LSPR peak centered at 520 nm. The absorption peak of FSA-20 experiences a red shift compared to Au nanocrystals. This phenomenon can be attributed to the change in the charge of Au nanocrystals induced by the dielectric interface provided by the SiO_2_ layer, which in turn triggers plasmon resonance [36]. With the increasing addition of Au, as depicted in Figure 2c, the absorption peak position of Fe_3_O_4_@SiO_2_-Au NCs undergoes a gradual red shift. It is primarily attributed to the decrease in spacing between adjacent Au nanocrystals on Fe_3_O_4_@SiO_2_ surfaces, leading to an enhanced plasmonic coupling effect [37].

**Figure 3:**
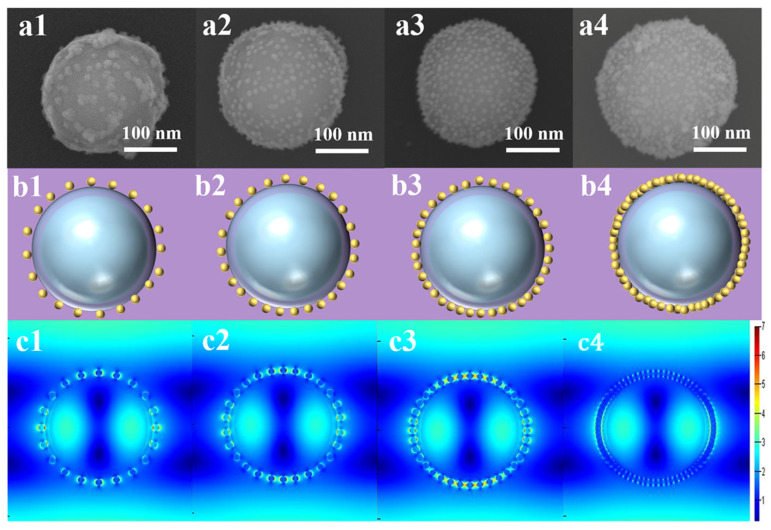
SEM image of (**a1**) FSA-20, (**a2**) FSA-40, (**a3**) FSA-60, (**a4**) FSA-80, and the corresponding (**b1**–**b4**) schematic diagrams and (**c1**–**c4**) electric field distribution simulation results.

**Figure 4:**
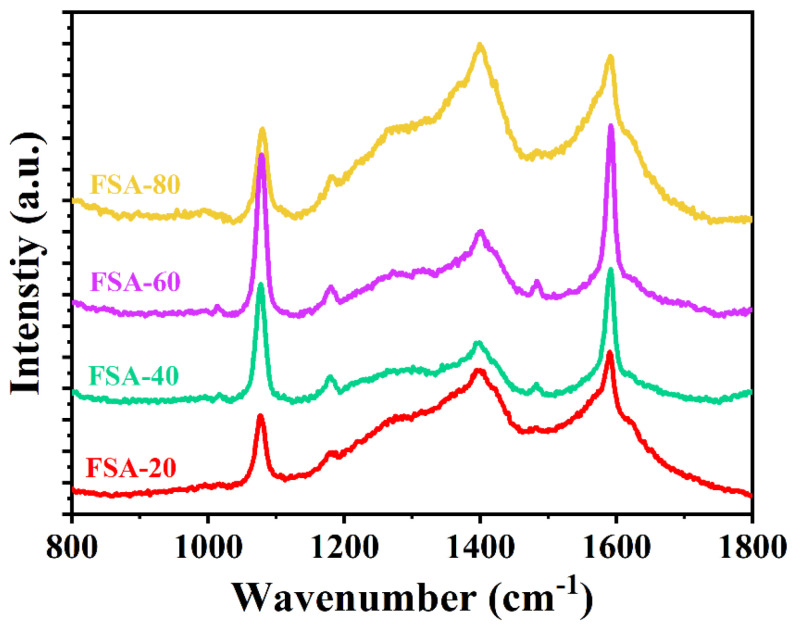
SERS spectra of 4-MBA (10^−4^ M) absorbed on FSA-20, FSA-40, FSA-60, and FSA-80.

Next, to analyze the effect of nanogap size between adjacent Au nanocrystals on SERS performance, we simulated the electric field distribution around Fe_3_O_4_@SiO_2_-Au NCs (FSA-20, FSA-40, FSA-60, and FSA-80) using the FDTD method. SEM images of FSA-2, FSA-4, FSA-6, and FSA-8 are shown in Figure 3a1–a4. It can be observed that the particle spacing of FSA-20, FSA-40, FSA-60, and FSA-80 is about 24, 16, 6, and 0 nm, respectively. The schematic diagrams (Figure 3b1–b4) and local electric field distribution (Figure 3c1–c4) of FSA-20, FSA-40, FSA-60, and FSA-80 are investigated. The simulation outcomes show that the predominant generation of high-intensity local electric fields occurs primarily within the nanogaps situated between Au nanocrystals. As the spacing between Au nanocrystals decreases, the local electric field exhibits a phenomenon of first increasing and then decreasing. This is because when the amount of Au colloid solution added increases to 80 mL, it may cause Au nanocrystals absorbed on the surfaces of Fe_3_O_4_@SiO_2_ to agglomerate and even form a Au shell, thereby reducing the number of SERS hotspots [38]. To further verify the variation trend in SERS performance with Au loading on the Fe_3_O_4_@SiO_2_ surfaces, we tested SERS spectra of 4-MBA (10^−4^ M) on different SERS substrates in Figure 4 [39]. The main Raman peak of 4-MBA at 1580 cm^−1^ can be clearly detected on SERS substrates with different Au loadings. With the increase in Au loading, SERS peak intensity at 1580 cm^−1^ first increases and then decreases, among which FSA-60 has the strongest SERS signal, which is consistent with the FDTD simulation results. It has been proven that FSA-60 has the optimal SERS sensitivity and is used as the following SERS-active substrate.

### 2.2. Capture and Detection of S. aureus by Apt-FSA-60

To selectively bind FSA-60 to *S. aureus*, SH-Apt was loaded onto their surfaces to form Apt-FSA-60, thus endowing them with specific recognition capabilities [40]. The specific binding ability of Apt-FSA-60 to target bacteria was verified through the following experiments. Initially, the *S. aureus* solution with different concentrations was standardized by utilizing PBS buffer. Subsequently, 1 mg of Apt-FSA-60 was immersed in 1 mL of *S. aureus* solution for 30 min. Meanwhile, FSA-60 was subjected to the same experimental procedure for comparison. Using magnets to enrich Apt-FSA-60, it can be observed from Figure S1a that the supernatant solution becomes clear, indicating that Apt-FSA-60 captured a large number of bacteria. As a control, FSA-60 without aptamer modification cannot capture bacteria, as the optical density of its supernatant is the same as that of the original *S. aureus* solution. Figure S1b displays the SEM image of the Apt-FSA-60-*S. aureus* complex, indicating that Apt-FSA-60 is tightly bound outside the *S. aureus*. To evaluate the specificity of Apt-FSA-60 to target *S. aureus*, four of the most common pathogens in clinics including *Escherichia coli*, *Salmonella typhimurium*, *Bacillus cereus*, and *Vibrio parahaemolyticus* were chosen as the unspecific bacteria. All the optical densities (ODs) of bacterial samples were set to 1. From Figure S2, it can be seen that the OD value of *S. aureus* is significantly lower than that of other interfering bacteria, which proves that Apt-FSA-60 is specifically capable of *S. aureus*.

**Figure 5:**
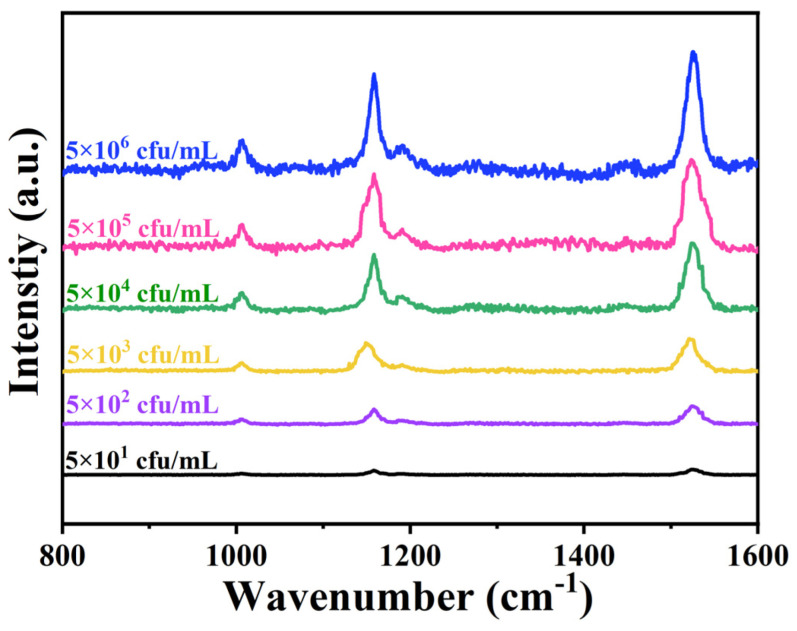
SERS spectra of *S. aureus* at concentrations of 5 × 10^1^ to 5 × 10^6^ cfu/mL.

Then, we conducted SERS detection on *S. aureus* at concentrations ranging from 5 × 10^1^ to 5 × 10^6^ cfu/mL based on Apt-FSA-60. The SERS spectra are shown in Figure 5. *S. aureus* has three different Raman peaks at 1004, 1158, and 1527 cm^−1^, which are indexed to phenylalanine, C-C, and C=C stretching vibration of β-carotene [41]. It is evident that Apt-FSA-60 can detect *S. aureus* with a low detection limit of 50 cfu/mL.

### 2.3. Investigation of Photothermal Performance of Apt-FSA-60

**Figure 6:**
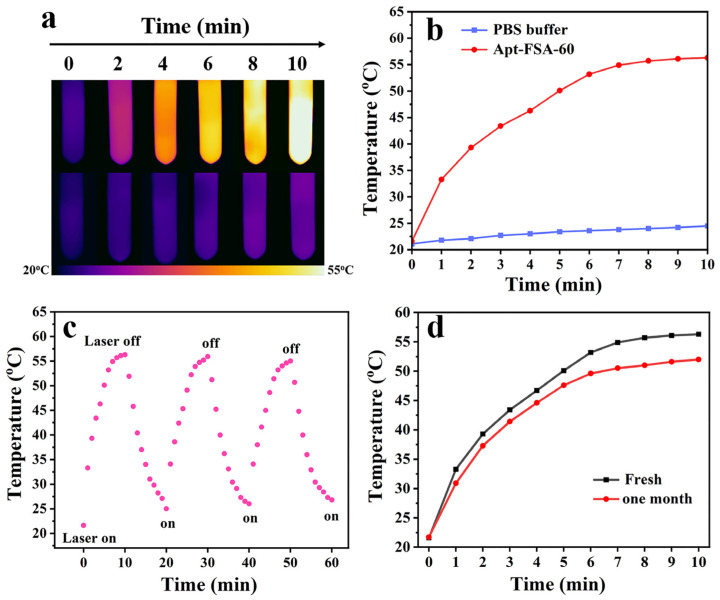
(**a**) IR thermal images and (**b**) photothermal heating curves of PBS buffer and Apt-FSA-60; (**c**) temperature curves of Apt-FSA-60 solution irradiated by 808 nm laser and passively cooled for three on/off cycles; (**d**) temperature variation curve of Apt-FSA-60 before and after one month of storage.

Next, we studied the photothermal performance of Apt-FSA-60. Apt-FSA-60 (200 μg/mL) was irradiated with NIR light, and the temperature change in real time was recorded by an IR thermal camera. As a control, the infrared thermal images of the temperature variation of the PBS solution over time were also tested. In Figure 6a, the temperature of the Apt-FSA-60 solution increases with the increase in irradiation time, while the temperature of the PBA buffer as a control sample remains almost unchanged. The corresponding temperature-over-time curve is observed in Figure 6b. It can be clearly seen that the temperature of Apt-FSA-60 increases by 34.7 °C within 10 min, while the PBS solution only observed a small temperature rise of about 3.4 °C under the same treatment, indicating that Apt-FSA-60 has good photothermal conversion ability. Figure 6c depicts the temperature variation curves of Apt-FSA-60 during three laser on/off cycles, and there is no significant change in the temperature, indicating that Apt-FSA-60 has excellent photothermal stability. The photothermal storage stability of the material was also investigated in Figure 6d. After being stored for one month, the temperature of Apt-FSA-60 increased by 30.4 °C under the same irradiation conditions, indicating its excellent photothermal storage stability.

### 2.4. PTT of S. aureus In Vitro

**Figure 7:**
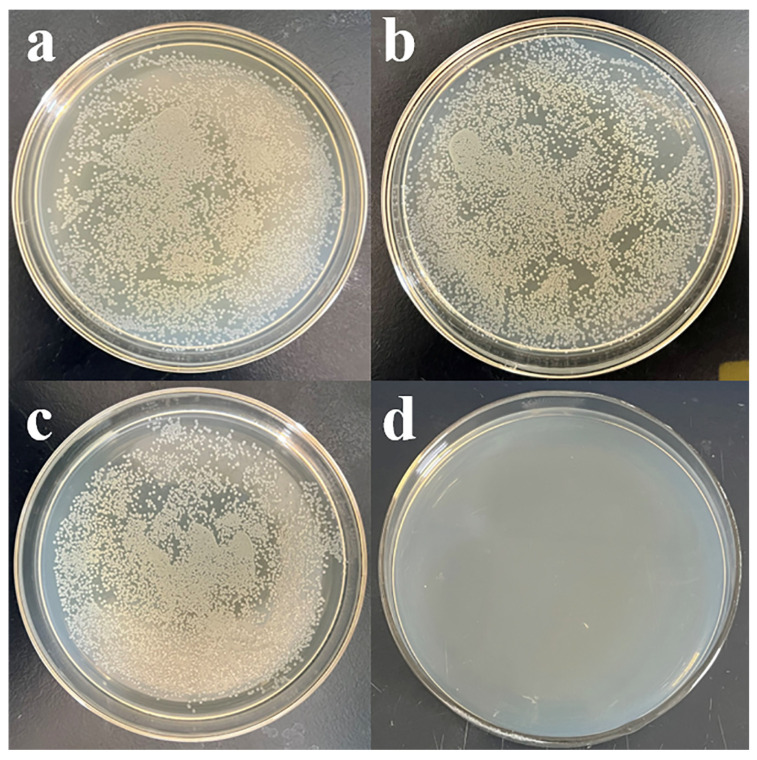
Photographs of plated bacterial colonies after treatment with (**a**) nothing; (**b**) only NIR laser; (**c**) only Apt-FSA-60; and (**d**) both NIR laser and Apt-FSA-60.

To explore the PTT inactivation behavior of the material against *S. aureus*, we conducted in vitro antibacterial experiments. The in vitro sterilization activity was studied through a plate counting experiment. Firstly, *S. aureus* bacterial suspension was diluted using PBS buffer and incubated with Apt-FSA-60, and then the mixture was irradiated with NIR laser for 10 min. Subsequently, three control experiments were conducted, including untreated *S. aureus* suspension, *S. aureus* suspension treated only with 808 nm laser, and *S. aureus* suspension incubated only with Apt-FSA-60. Subsequently, four groups of bacterial suspensions were evenly spread on agar plates and incubated for 24 h at 37 °C. As shown in Figure 7, it can be observed that bacterial cells grew well on the agar plates of groups (a) to (c), while only sporadic bacterial colonies appeared in group (d). This indicates that the superior photothermal effect of Apt-FSA-60 is fully sufficient to cause thermal damage to *S. aureus*.

**Figure 8:**
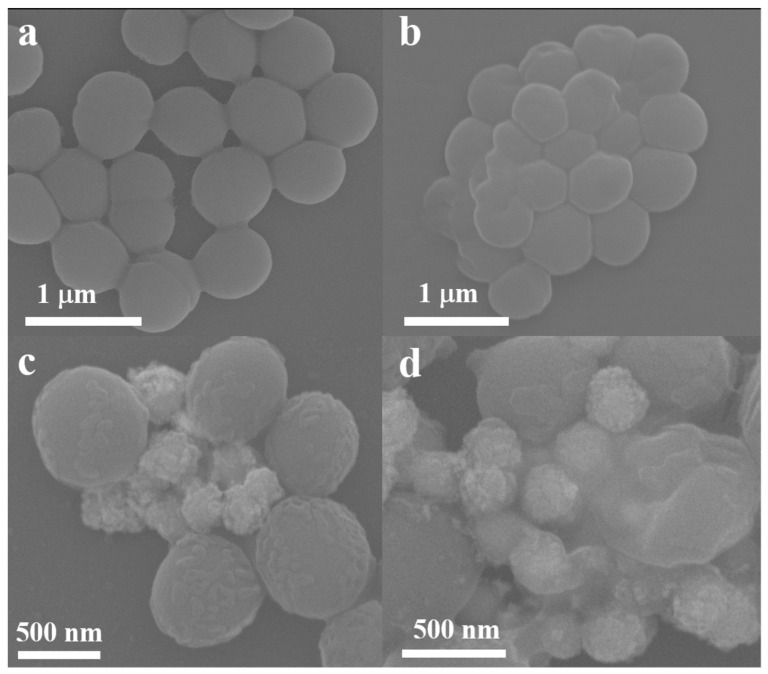
SEM images of *S. aureus* after treatment with (**a**) nothing; (**b**) only NIR laser; (**c**) only Apt-FSA-60; and (**d**) both NIR laser and Apt-FSA-60.

Moreover, to gain a deep understanding of the antibacterial mechanism of Apt-FSA-60, an SEM test was used to study the morphological changes in *S. aureus* after different treatment methods [42]. As shown in Figure 8a, the untreated *S. aureus* typically appears spherical, with a smooth and intact cell membrane surface. After treating *S. aureus* with NIR laser (Figure 8b) or Apt-FSA-60 (Figure 8c), a minor degree of damage can be detected on the surface of the bacterial membrane, which demonstrates that treatment with either the NIR laser or the material alone has little to no impact on bacterial activity. By comparison, after simultaneously using an 808 nm laser and Apt-FSA-60 to treat *S. aureus*, the bacterial cell membrane ruptures and collapses, completely losing its normal shape, as can be seen in Figure 8d. The above results demonstrate that the bactericidal effect of Apt-FSA-60 is caused by the disruption of cell membrane integrity, leading to bacterial lysis and death.

## 3. Materials and Methods

### 3.1. Preparation of Fe_3_O_4_@SiO_2_-Au NCs

Fe_3_O_4_ nanocrystals were prepared by the simple solvothermal method. A total of 1 g of FeCl_3_·6H_2_O, 2.8 g of NaAc, 0.32 g of Na_3_C_6_H_5_O_7_·2H_2_O, and 0.16 g of PEG were sequentially dissolved in 56 mL of EG solution and then continue stirred until completely dissolved. Subsequently, the mixture was transferred to a three-neck flask and heated to 160 °C under mechanical stirring. After 50 min, the solution was quickly transferred to an autoclave and continuously heated for 9 h at 180 °C. Finally, the product was collected using a magnet and rinsed several times to obtain Fe_3_O_4_ nanocrystals.

Fe_3_O_4_@SiO_2_ nanocrystals were prepared using the sol-gel method. A total of 100 mg of Fe_3_O_4_ nanocrystals was dispersed in a water-ethanol mixed solution. It was then subjected to ultrasonic treatment for 10 min until evenly mixed. Afterward, 2 mL of NH_3_·H_2_O solution was added, and the ultrasonic treatment was continued for 2 h. Then, 100 μL of TEOS was added, and the mixture was mechanically stirred overnight to obtain Fe_3_O_4_@SiO_2_ nanocrystals.

As for the synthesis process of Fe_3_O_4_@SiO_2_-Au NCs, the obtained Fe_3_O_4_@SiO_2_ nanocrystals were dissolved in 40 mL of ethanol. A total of 0.5 mL of APTES was then added dropwise. The above solution was mechanically stirred to obtain Fe_3_O_4_@SiO_2_-NH_2_. Meanwhile, the Au colloidal solution was synthesized using the sodium citrate reduction method [43]. Subsequently, 50 mL of the Au colloid solution was added, and the mixture was continuously sonicated until the solution became discolored to form Fe_3_O_4_@SiO_2_-Au NCs. Fe_3_O_4_@SiO_2_-Au NCs with different Au colloid solution addition amounts of 20, 40, 60, and 80 mL were named FSA-20, FSA-40, FSA-60, and FSA-80, respectively.

### 3.2. Preparation of Apt-FSA-60

To activate the SH-Apt for *S. aureus*, 10 μL of SH-Apt (10^−5^ M) was added into 10 μL of TCEP solution (1 mM). Then, the activated SH-Apt was added to FSA-60 and placed in a thermostatic oscillator for agitation overnight at 37 °C. Finally, the Apt-FSA-60 product was collected using a magnet, and its surface was rinsed with PBS buffer to remove any unattached aptamers.

### 3.3. S. aureus Sample Preparation

Firstly, 4 g of Luria-Bertani (LB) agar medium powder was dissolved in 100 mL of ultrapure water and stirred evenly. Subsequently, the culture medium and culture dish were transferred to the autoclave at 121 °C for high-pressure sterilization treatment. After 15 min, the sterilized culture medium was naturally cooled at room temperature. After cooling to 50 °C, the culture medium was poured into a sterile and dry culture dish. Finally, *S. aureus* strains stored in an environment of −20 °C were removed and inoculated onto the culture medium, and then, they were grown in a constant temperature incubator at 37 °C overnight.

### 3.4. SERS Detection of S. aureus

Different concentrations of *S. aureus* were incubated with Apt-FSA-60 in a thermostatic oscillator for 30 min at 37 °C. After collecting the Apt-FSA-60-*S. aureus* complex with a magnet, it was rinsed with PBS three times and placed on a slide for further SERS detection.

### 3.5. Photothermal Conversion Performance of Apt-FSA-60 NCs

The photothermal conversion performance of Apt-FSA-60 was tested by continuously irradiating Au nanocrystals and Apt-FSA-60 with NIR laser for 10 min. Temperature images of the solution were captured every minute using an IR thermal imaging camera. To explore the photostability of Apt-FSA-60, the solution was allowed to cool naturally for 10 min after irradiation, with a total of three cycles.

### 3.6. Incubation of S. aureus In Vitro

Under an 808 nm laser, *S. aureus* was incubated with Apt-FSA-60 at 37 °C for 30 min, followed by irradiation with an 808 nm laser for 10 min. Then, three parallel experiments, including *S. aureus* before and after laser treatment and *S. aureus* incubated with Apt-FSA-60, were conducted. Finally, the four groups of bacterial solution were poured into four LB agar plates for incubation to directly observe the survival of bacteria.

### 3.7. Morphology of the Treated S. aureus Determined by SEM

The SEM test was performed to study the size and morphological changes in bacteria under different conditions. After washing *S. aureus* with PBS buffer and redispersing it in PBS, the bacterial suspension was incubated with Apt-FSA-60 at 37 °C for 30 min. Subsequently, the mixture was irradiated for 10 min under NIR laser. Apt-FSA-60-bacteria complex was collected using a magnet, and the sample was treated with 2.5% glutaraldehyde. After fixation, the complex was washed with PBS buffer and dehydrated stepwise for 10 min each in 30%, 50%, 70%, 85%, 90%, and 100% ethanol. Finally, the sample was dropped onto a silicon substrate for SEM testing.

## 4. Conclusions

In summary, a novel multifunctional Apt-Fe_3_O_4_@SiO_2_-Au NCs was successfully synthesized and can be used for the selective capture, SERS detection, and photothermal inactivation of *S. aureus*. The relationship between the nanogap between adjacent Au nanocrystals and SERS performance was studied by changing the amount of Au nanocrystals on Fe_3_O_4_@SiO_2_ surfaces. The results indicated that as the nanogap between Au nanocrystals decreased, the SERS intensity of 4-MBA first increased and then decreased, depending on the number of hotspots in the SERS substrate. Then, combining the optimal FSA-60 SERS substrate with the aptamer could achieve the specific and sensitive detection of *S. aureus*, with a detection limit of 50 cfu/mL. Furthermore, under 808 nm laser irradiation, Apt-FSA-60 showed excellent photothermal properties and may effectively kill *S. aureus* within 10 min. The changes in the surface morphology of *S. aureus* observed in SEM images indicated that the bacterial death was caused by the high temperature generated by Apt-FSA-60, which damaged the bacterial cell membrane. The developed multifunctional Apt-FSA-60 provides a novel approach for the monitoring and killing of foodborne pathogens and has broad application value in clinical diagnosis and treatment.

## Data Availability

The data that support the findings of this study are available from the corresponding authors upon reasonable request.

## Supplementary Materials

The following supporting information can be downloaded at https://www.mdpi.com/article/10.3390/molecules29153593/s1: Figure S1: Photo images of (a) *S. aureus* solution, FSA-60-*S. aureus* complex solution after magnetic collection (control), and Apt-FSA-60-*S. aureus* complex after magnetic collection; (b) SEM image of Apt-FSA-60-*S. aureus* complex; Figure S2: OD600 value of supernatant solution of different bacteria after being incubated with Apt-FSA-60, (a–e): *Escherichia coli*, *Salmonella typhimurium*, *Bacillus cereus*, *Vibrio parahaemolyticus,* and *S. aureus*.
