# Supplementary material for: Controllable Construction of Aptamer-Modified Fe3O4@SiO2-Au Core-Shell-Satellite Nanocomposites with Surface-Enhanced Raman Scattering and Photothermal Properties and Their Effective Capture, Detection, and Elimination of Staphylococcus aureus
Source: Molecules. 2024 Jul 30;29(15):3593. doi: 10.3390/molecules29153593 (PMC11314609; doi:10.3390/molecules29153593)
Supplement: Supplementary file 1 [file molecules-29-03593-s001.zip › molecules-3060705-supplementary.pdf]

Controllable Construction of Aptamer-Modified Fe_3O_4@SiO_2-Au Core-Shell-Satellite Nanocomposites with Surface-enhanced Raman Scattering and Photothermal Properties and Their Effective Capture, Detection, and Elimination of *Staphylococcus aureus*

**Yongdan Wang [1,2], Shengyi Wang [2], Yuhui Zou [1], Yuze Gao [2], Boya Ma [2], Yuhan Zhang [2], Huasong Dai [2],
Jingmei Ma [2] and Wenshi Zhao [2,*]**

[1] School of Foreign Languages, Jilin Normal University, Siping 136000, China; ydwang@jlnu.edu.cn (Y.W.); 15898157806@163.com (Y.Z.)
[2] Key Laboratory of Functional Materials Physics and Chemistry of the Ministry of Education, Jilin Normal University, Changchun 130103, China; 15943440659@163.com (S.W.); 19990599393@163.com (Y.G.); 13664198753@163.com (B.M.); zhangyuhan193@163.com (Y.Z.); 15948835770@163.com (H.D.); jlsfdxmjm@163.com (J.M.)
* Correspondence: 2405006@jlnu.edu.cn

Materials

Iron(III) chloride hexahydrate ($FeCl_3·6H_2O$), Sodium acetate (NaAc), sodium citrate dihydrate ($Na_3C_6H_5O_7·2H_2O$), polyethene glycol (PEG), ethylene glycol (EG), phosphate buffer solution (10 mM PBS, pH 7.4), glutaraldehyde, tris-(2-carboxyethyl) phosphine hydrochloride (TCEP) and ethanol were procured from Sinopharm Chemical Reagent Co., Ltd. Ammonia aqueous solution ($NH_3·H_2O$) and tetraethyl orthosilicate (TEOS) were obtained from Shanghai Macklin Biochemical Co., Ltd. 3-aminopropyl triethoxysilane (APTES), 4-mercaptobenzoic acid (4-MBA) and gold chloride trihydrate ($HAuCl_4·3H_2O$) were obtained from Aladdin Reagent Co., Ltd. (Shanghai, China). All chemicals utilized in the experiment were of analytical grade, ensuring utmost precision. Additionally, ultrapure water, purified through the Milli-Q system, was consistently employed throughout the experimental process.

Biochemicals

S. aureus strains were obtained from MiaoLing Plasmid Sharing Platform (Wuhan, China). and their thiolate aptamer (5′-SH-GCA ATG GTA CGG TAC TTC CTC GGC ACG TTC TCA GTA GCG CTC GCT GGT CAT CCC ACA GCT ACG TCA AAA GTG CAC GCT ACT TTG CTA A-3′) was bought from Shanghai Sangon Biological Science & Technology Company (Shanghai, China).

Apparatus

The structure, morphologies and elemental compositions of the prepared samples were tested by transmission electron microscope (TEM; JEOL 2100), scanning electron microscopy (SEM) (JEOL JSM-7800F), energy dispersive spectrometer (EDS) analysis (JEOL Ltd, Tokyo, Japan) and X-ray diffractometer (XRD) (XRD; Rigaku D/Max-2500) using Cu Kα radiation (λ =1.5406 Å). Shimadzu UV 3600 spectrophotometer (Shimadzu Corporation, Japan) and quantum design MPMS3 superconducting quantum interference device (SQUID) magnetometer (Quantum Design, Inc., USA) were used to measure the optical and magnetic properties of samples. SERS spectra were recorded in the region from 800 to 1800 cm^{-1} by Renishaw inVia Raman spectrometer (Renishaw, London, UK) equipped with a 632.8 nm wavelength. An 808nm multimode laser diode (LR-MFJ-808/2000 mW) was used for the temperature rise experiment, and the temperature changes were recorded using an infrared thermal imaging camera (FOTRIC 326C-L44, China).

Figure S1

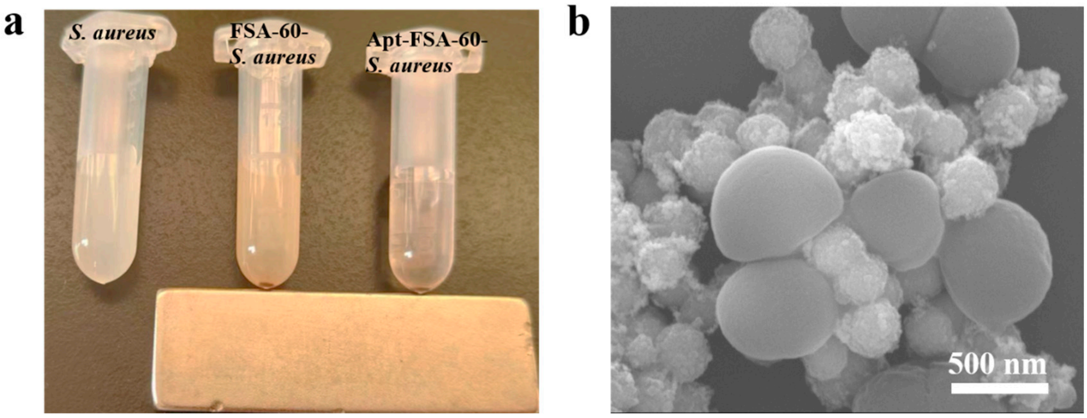

Figure S1. Photo images of (a) *S. aureus* solution, FSA-60-*S. aureus* complex solution after magnetic collection (control), and Apt-FSA-60-*S. aureus* complex after magnetic collection, (b) SEM image of Apt-FSA-60-*S. aureus* complex.

Figure S2

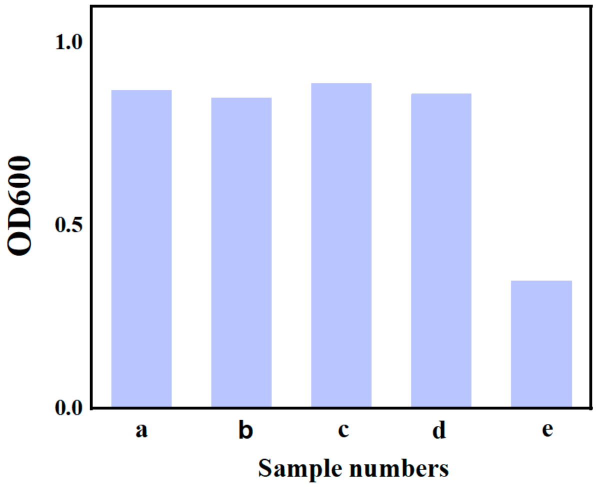

Figure S2. OD600 value of supernatant solution of different bacteria after being incubated with Apt-FSA-60, a-e: *Escherichia coli*, *Salmonella typhimurium*, *Bacillus cereus*, *Vibrio parahaemolyticus* and *S. aureus*.
